# Modeling and Experimental Validation of Cell Morphology in Microcellular-Foamed Polycaprolactone

**DOI:** 10.3390/polym16192723

**Published:** 2024-09-26

**Authors:** Donghwan Lim, Sanghyun Lee, Seungho Jung, Kwanhoon Kim, Jin Hong, Sung Woon Cha

**Affiliations:** 1 Department of Mechanical Engineering, Yonsei University, 50 Yonsei-ro, Seodaemun-gu, Seoul 03722, Republic of Korea; imdh@yonsei.ac.kr (D.L.); kimkevin99@yonsei.ac.kr (K.K.); jin.hong@yonsei.ac.kr (J.H.); 2 School of Mechanical Engineering, Sungkyunkwan University, Suwon 16419, Republic of Korea; davincilee@g.skku.edu; 3 Department of Mechanical Engineering, Korea Advanced Institute of Science and Technology (KAIST), 291 Daehak-ro, Yuseong-gu, Daejeon 34141, Republic of Korea; hifivejsh@kaist.ac.kr

**Keywords:** microcellular foaming, cell morphology, biopolymer, nucleation theory

## Abstract

This study investigates the modeling and experimental validation of cell morphology in microcellular-foamed polycaprolactone (PCL) using supercritical carbon dioxide (scCO_2_) as the blowing agent. The microcellular foaming process (MCP) was conducted using a solid-state batch foaming process, where PCL was saturated with scCO_2_ at 6 to 9 MPa and 313 K, followed by depressurization at a rate of −0.3 and −1 MPa/s. This study utilized the Sanchez–Lacombe equation of state and the Peng–Robinson–Stryjek–Vera equation of state to model the solubility and density of the PCL-CO_2_ mixture. Classical nucleation theory was modified and combined with numerical analysis to predict cell density, incorporating factors such as gas absorption kinetics, the role of scCO_2_ in promoting nucleation, and the impact of depressurization rate and saturation pressure on cell growth. The validity of the model was confirmed by comparing the theoretical predictions with experimental and reference data, with the cell density determined through field-emission scanning electron microscopy analysis of foamed PCL samples. This study proposes a method for predicting cell density that can be applied to various polymers, with the potential for wide-ranging applications in biomaterials and industrial settings. This research also introduces a Python-based numerical analysis tool that allows for easy calculation of solubility and cell density based on the material properties of polymers and penetrant gases, offering a practical solution for optimizing MCP conditions in different contexts.

## 1. Introduction

The first polymeric foam was proposed in the 1930s through a patent for producing polystyrene foams (1931). This foam consisted of cells larger than 100 μm in size. Additionally, due to the non-uniformity in cell size, the mechanical properties were reduced, making it insufficient for practical use in structural or mechanical materials. Later, in the 1980s, the microcellular foaming process (MCP) was developed and advanced at the Massachusetts Institute of Technology (MIT) through the solid-state batch foaming process [1]. The solid batch foaming process is an important experimental method in the laboratory, where the solid-state polymer is placed in a batch chamber to observe the behavior and physical phenomena of microcell formation. The development of the MCP has led to significant advancements in the field of polymer processing. It has been widely applied in various areas, resulting in reduced material consumption, improved mechanical properties such as tensile strength and impact strength, and enhanced thermal and electrical insulation performance [2,3,4]. Microcellular foam is defined as having cells with a size of less than 10 microns and a cell density of over 10^9^ cells/cm^3^ [5,6,7]. The MCP is created by saturating a polymer with gas under high pressure, followed by pressure release or heating to induce thermodynamic instability, which in turn triggers the nucleation and growth of cells. During the foaming process using the MCP, supercritical carbon dioxide (scCO_2_) is commonly employed as an effective method for gas saturation. scCO_2_ possesses the density of a liquid and the viscosity of a gas. scCO_2_ is achieved at pressures above 7.4 MPa and temperatures above 304 K. Additionally, it is predominantly used in the foaming process because it does not leave residual solvent issues. Moreover, using scCO_2_ as a blowing agent offers advantages such as high diffusivity, low surface tension, and environmentally friendly characteristics [8,9,10].

Polycaprolactone (PCL) has attracted attention in the fields of biomedical engineering and environmentally friendly materials due to its biodegradability and biocompatibility [11]. Numerous drug delivery systems made from PCL have already been approved by the U.S. Food and Drug Administration and certified with a CE mark, making them appropriate for use as implantable medical devices in humans [12,13,14]. The combination of PCL and scCO_2_ enables the production of bio-based microcellular foam with a desired cell morphology. If cell morphology can be freely controlled, the in vivo biodegradation time of foamed PCL can be intentionally adjusted [15]. Cell morphology, including cell density and cell size, is largely influenced by the precise control of various process parameters such as the amount of gas absorption, thermal stimuli, and depressurization rate.

Given the various factors influencing cell morphology, this study aims to model the cell structure of PCL foam generated using scCO_2_ and analyze the impact of the depressurization rate, which is one of the most easily controllable parameters, on cell density and size. In addition, the theoretical modeling will be validated by comparing the model predictions, developed by combining classic nucleation theory (CNT), the Sanchez–Lacombe equation of state (SL-EOS), and a chemical equilibrium model with experimental data. Recent studies have utilized computer-based analytical tools for structural prediction and target identification as primary research methods [16,17]. While theoretical models for cell nucleation and growth in polymer foaming are well established, applying these models to the PCL and scCO_2_ system requires identifying and incorporating the unique interactions of this specific system, which presents certain challenges.

Several key studies have laid an important foundation for understanding the mechanisms of nucleation and growth in polymer microcellular foaming. Colton and Suh (1987) presented a comprehensive model for the nucleation of thermoplastic microcellular foam, including the effects of the type of blowing agent and gas solubility on nucleation [5,18]. Recent advancements have extended these models to account for the thermodynamics and diffusivity of gases within polymers under supercritical conditions. For instance, Cotugno et al. (2005) [19] and Karimi et al. (2011) [20] studied the thermodynamics and mutual diffusivity of CO_2_ in molten PCL, providing critical parameters necessary for modeling gas–polymer interactions. However, modeling cell morphology resulting from the interaction between melted PCL and scCO_2_ differs from the behavior of cell morphology in un-melted PCL and scCO_2_. This is because, in order to create high-value products such as biomaterials or medical devices using the solid batch foaming process, it is essential to accurately understand the behavior of solid-state PCL and scCO_2_. Therefore, gas absorption and cell morphology were analyzed below the melting point of PCL to ensure that the PCL remained solid throughout the entire foaming process. Therefore, based on these foundational studies, this paper developed an accurate model for the cell morphology of PCL foam produced using scCO_2_, focusing on the effects of depressurization rate and saturation pressure. The model includes the kinetics of gas absorption, the role of scCO_2_ in promoting nucleation, and the impact of the depressurization rate on cell growth. By integrating experimental observations with theoretical predictions, we established correlations that enhance the understanding of the MCP and proposed MCP conditions that can create the desired cell morphology for various applications.

## 2. Materials and Methods

### 2.1. Materials

**Figure 1:**
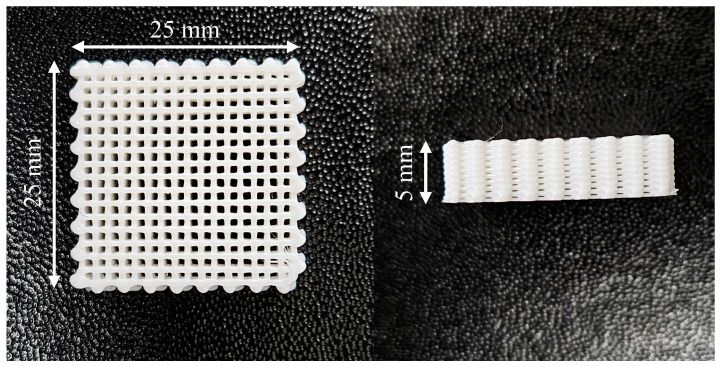
Optical image of 3D-printed PCL specimen and size.

A PCL filament (eMate PCL, eSUN, Shenzhen, China) was prepared as the polymer matrix for 3D printing and MCP. PCL molecular weights range from 3000 to 90,000 g/mol [21,22]. PCL is semi-crystalline, biodegradable, and biocompatible with a density of 1.16 g/cm^3^, a glass transition temperature (*T_g_*) of 213 K, and a melting temperature (*T_m_*) of 333 K. Since it can be used as a material for medical devices and biodegradable implants through three-dimensional (3D) printing, the MCP was applied to 3D printed specimens. Given that each individual’s organs and internal structures are different, 3D printing was employed to create optimally shaped medical devices that are precisely designed to perform their intended functions. A 3D printer (X1-Carbon Combo; Bambu Lab, Shenzhen, China) was used to make a sample specimen (Figure 1). The parameters of 3D printing are below. The nozzle and bed temperatures are 443 K and 313 K. The nozzle diameter is 0.4 mm, with a printing speed of 15 mm/s. The layer thickness is 0.25 mm. The infill pattern was set to a 50% grid pattern to absorb gas faster than the solid type of PCL. In the MCP, CO_2_ (99.9%; 40 L; Samheung GasTech, Seoul, Republic of Korea) was utilized as a blowing agent to ensure stable saturation and high solubility.

### 2.2. Microcellular Foaming Process

**Figure 2:**
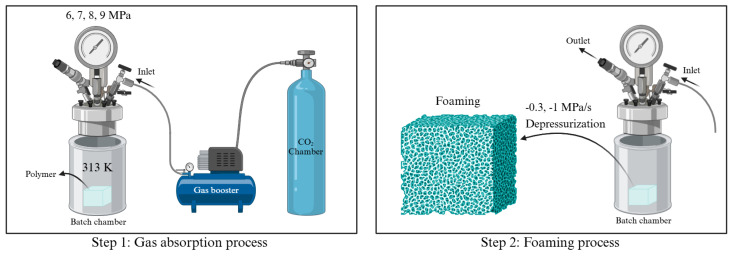
Overall process of microcellular foaming process (created with BioRender.com).

To ensure the stable production of the MCP, a solid-state batch foaming process was used. The MCP is carried out in two steps, as shown in Figure 2: the gas absorption process and the foaming process. This involved placing the polymer in a chamber, increasing the pressure up to 6~9 MPa using a gas booster (GB-SS, Pumster, Daejeon, Republic of Korea), and heating the batch chamber to 313 K with a band heater to create a scCO_2_ state. The polymer was then saturated with gas under these conditions. To investigate the interaction between CO_2_ and the solid-state polymer, gas absorption was conducted at 313 K, a temperature below the melting point of PCL (333 K). Figure 2 provides a schematic overview of the MCP using a batch chamber system. Considering that the *T_g_* of PCL is around 213 K, microcellular foam forms inside the chamber during depressurization [23,24,25]. The depressurization rate was controlled by adjusting the opening of the outlet valve to achieve the desired rate. Two ball valves were utilized to regulate the depressurization process. One valve was partially opened to enable controlled pressure release, while the other was fully opened to expedite the process. The time required for the pressure to reach atmospheric levels was carefully measured, allowing for the consistent achievement of a depressurization rate of −0.3 and −1 MPa/s. This approach provided precise control over the depressurization process, ensuring the repeatability and reliability of the experimental conditions. More detailed MCP conditions are provided in Table 1.

### 2.3. Modeling

#### 2.3.1. Thermodynamic Analysis to Predict Weight Fraction of CO_2_

Thermodynamic analysis was employed to model and predict the solubility, defined as the amount of scCO_2_ saturated in PCL within the batch chamber. The modeling leveraged the SL-EOS to characterize the state of the PCL–CO_2_ mixture during solid-state gas absorption [26,27]. The SL-EOS enables accurate predictions in both subcritical and supercritical fluid states. However, relying solely on the SL-EOS to describe the system can result in reduced accuracy due to insufficient information. To address this, additional modeling was conducted by incorporating an equation. It was assumed that the chemical potential of CO_2_ in the PCL–scCO_2_ mixture equals the chemical potential of pure CO_2_ at equilibrium (leveraging the concept of chemical potential equilibrium).

Additionally, due to the variability of CO_2_ density with temperature and pressure, this factor was continuously integrated into the equations to improve the accuracy of system predictions. To achieve this, a modified Peng–Robinson equation of state, specifically the Peng–Robinson–Stryjek–Vera equation of state (PRSV-EOS), was employed. The PRSV-EOS is known for its accuracy in predicting CO_2_ density as a function of pressure and has been validated against experimental data [28].

(1) $$P=\frac{RT}{\left(v-b\right)}-\frac{a}{\left(v\left(v+b\right)+b\left(v-b\right)\right)}$$

(2) $$a=\alpha (0.457235{\left(RT_{c}\right)}^{2})/P_{c}$$

(3) $$b=0.077796\;RT_{c}/P_{c}$$

(4) $$\alpha ={\left(1+\kappa (1-{T_{c}}^{0.5}\right)}^{2}$$

(5) $$T_{r}=T/T_{c}$$

(6) $$\kappa =\kappa_{0}+\kappa_{1}(1+{T_{c}}^{0.5})(0.7-T_{r}),\;when\;T_{r}\leq 0.7$$

(7) $$\kappa_{0}=0.378893+1.4897153\omega -0.17131848\omega^{2}+0.0196544\omega^{3},\;when\;T_{r}>0.7$$

The density of CO_2_ was predicted using the PRSV-EOS, as outlined in Equations (1)–(7). The parameters necessary for the PRSV-EOS are summarized in Table 2. Using these parameters, the density of CO_2_ at 313 K can be computed as a function of pressure. This enables accurate predictions of CO_2_ density across all temperatures and pressures in both subcritical and supercritical states.

In the case of PCL, foaming occurred immediately after the depressurization following gas absorption. Therefore, when scCO_2_ was used, accurately determining the weight gain of the gas proved challenging without employing a specialized balance (such as a quartz balance), which did not absorb the gas. The validity of the model was confirmed by comparing it with the solubility measurements reported in other studies [29]. To calculate the density of the PCL–CO_2_ mixture, we used the SL-EOS, as shown in Equation (8). Equation (8) describes the state equation for the density of the PCL–CO_2_ mixture with dissolved CO_2_. It incorporates characteristic pressure ($P^{*}$), characteristic density ($\rho^{*}$), characteristic temperature ($T^{*}$), reduced density ($\tilde{\rho }$), reduced pressure ($\tilde{P}$), and reduced temperature ($\tilde{T}$), with the relationship between these parameters detailed in Equation (9).

(8) $$\tilde{\rho }=1-exp\left[-\frac{{\tilde{\rho }}^{2}}{\tilde{T}}-\frac{\tilde{P}}{\tilde{T}}-\left(1-\frac{\phi_{1}}{r_{1}}\right)\tilde{\rho }\right]$$

(9) $${\tilde{P}}_{i}=\frac{P}{{P_{i}}^{*}},\;{\tilde{\rho }}_{i}=\frac{\rho }{{\rho_{i}}^{*}},\;{\tilde{T}}_{i}=\frac{T}{{T_{i}}^{*}}\;$$

Subscripts 1 and 2 represent CO_2_ and PCL, respectively, and the absence of a subscript denotes the PCL–CO_2_ mixture. $\phi_{1}$ denotes the volume fraction of CO_2_ dissolved in the PCL–CO_2_ mixture, while $r_{1}$ indicates the number of lattice sites occupied by CO_2_ molecules in the mixture. Table 3 lists the characteristic values of these parameters for CO_2_ and PCL, which were used in the SL-EOS to calculate the density of the PCL–CO_2_ mixture [19].

Equations (8)–(12) were used to determine the characteristic pressure and temperature of the PCL–CO_2_ mixture, as follows:

(10) $$P^{*}=\phi_{1}{P_{1}}^{*}+\phi_{2}{P_{2}}^{*}-\phi_{1}\phi_{2}{∆P}^{*}$$

(11) $${∆P}^{*}={P_{1}}^{*}+{P_{2}}^{*}-{2\psi ({P_{1}}^{*}{P_{2}}^{*})}^{0.5}$$

(12) $$T^{*}=\frac{P^{*}}{\frac{\phi_{1}{P_{1}}^{*}}{{T_{1}}^{*}}+\frac{\phi_{2}{P_{2}}^{*}}{{T_{2}}^{*}}}$$

The relationship between the volume fraction of CO_2_ and the reduced density of the mixture was determined using Equations (1)–(12). ψ is the binary interaction parameter for CO_2_ and PCL and is a dimensionless parameter used to calculate ${P_{12}}^{*}$ as the geometric mean. For the PCL–CO_2_ mixture, *ψ* was experimentally fitted to 0.98 to achieve accurate correlation [10]. The volume fraction of CO_2_ can be determined using the chemical potential equilibrium equation presented in Equation (13).

(13) $$\begin{array}{c} \left[-\frac{{\tilde{\rho }}_{1}}{T_{1}}+\frac{{\tilde{P}}_{1}}{{\tilde{T}}_{1}{\tilde{\rho }}_{1}}+\frac{\left(1-{\tilde{\rho }}_{1}\right)\ln\left(1-{\tilde{\rho }}_{1}\right)}{{\tilde{\rho }}_{1}}+\frac{ln{\tilde{\rho }}_{1}}{r_{1}}\right]r_{1}\\ =ln\phi_{1}+\left(1-\phi_{1}\right)+\tilde{\rho }X_{1}{\left(1-\phi_{1}\right)}^{2}\\ +\left[-\frac{\tilde{\rho }}{T_{1}}+\frac{{\tilde{P}}_{1}}{{\tilde{T}}_{1}\tilde{\rho }}+\frac{\left(1-\tilde{\rho }\right)\ln\left(1-\tilde{\rho }\right)}{\tilde{\rho }}+\frac{ln\tilde{\rho }}{r_{1}}\right]r_{1} \end{array}$$

$\chi_{1}$ in Equation (13) is expressed as

(14) $$\chi_{1}=\frac{{\Delta P}^{*}M_{1}}{RT{\rho_{1}}^{*}}$$

Next, the binary interaction parameter was determined using Equation (14), and the weight fraction of CO_2_ was calculated using Equation (15). This allowed for the final determination of CO_2_ solubility in PCL as a function of pressure.

(15) $$\omega_{1}=\frac{\phi_{1}}{\phi_{1}+(1-\phi_{1})\frac{{\rho_{2}}^{*}}{{\rho_{1}}^{*}}}$$

#### 2.3.2. Nucleation Theory

The CNT model describes the formation of homogeneous bubble nucleation by leveraging the relationship between various properties of the polymer and the interfacial tension between the polymer and gas [5]. Microcellular foams can be categorized into three types based on the nucleation process: homogeneous, heterogeneous, and mixed mode. These classifications depend on whether bubbles are uniformly generated due to thermodynamic instability in the CO_2_ mixture and on whether secondary cell nucleation occurs at the boundaries of the formed bubbles [18]. In this study, since uniform nucleation was experimentally confirmed, the modeling focused on homogeneous cell nucleation. The homogeneous nucleation rate is given by

(16) $$N_{hom}=Af_{0}C_{0}\exp(\frac{{-{\Delta G}^{*}}_{hom}}{kT}B)$$

Here, $f_{0}\;(s^{-1})$ represents the frequency factor for CO_2_, while *C*_0_ (number of molecules/cm^3^) denotes the concentration of gas molecules in the PCL–CO_2_ mixture. k (J/K) is the Boltzmann constant, and *T* (K) indicates the temperature. The parameter $f_{0}$ describes the rate at which nuclei with a critical radius convert into stable bubbles; its value for homogeneous cell nucleation was determined as 10^−5^ $s^{-1}$ [30,31,32,33]. *A* and *B* are correction factors that account for the different physical properties of various materials. Since the exact value of the frequency factor is not known, an A factor was added as a correction factor for the frequency factor. Although delta Gibbs energy can be calculated using the interfacial surface tension of the mixture, it is also very difficult to obtain an accurate value for this. Therefore, a B factor was introduced as a correction factor to complete the equation. In this study, the value of *A* and *B* for the PCL–CO_2_ mixture was fitted to 1.1 and 0.0095, respectively. *A* and *B* have different values depending on the material, but for the same material, they maintain the same values even with different depressurization rates. Additionally, ${{\Delta G}^{*}}_{hom}$ represents the homogeneous nucleation activation energy, as defined by Equation (18).

$\gamma_{mix}$ (expressed by Equation (17)) represents the surface tension of the PCL–CO_2_ mixture, while ΔP denotes the depressurization rate for PCL foaming. The interfacial surface tension of the PCL–CO_2_ mixture can be determined based on the relationship between the density of the PCL–CO_2_ mixture, the density of PCL, and the weight fraction of CO_2_ [24]. The surface tension of PCL ($\gamma_{PCL}$) used in this study was 0.0456 N/m, based on the prior literature [34].

(17) $$\gamma_{mix}=\gamma_{PCL}{\left(\frac{\rho_{mix}}{\rho_{PCL}}\right)}^{4}{\left(1-\omega_{CO_{2}}\right)}^{4}$$

(18) $${{\Delta G}^{*}}_{hom}=\frac{16\pi {\gamma_{mix}}^{3}}{3{\Delta P}^{2}}$$

$N_{hom}$ is calculated using Equation (16), based on ${{\Delta G}^{*}}_{hom}$ computed from Equation (18) and the concentration derived from the weight fraction of CO_2_. The cell density can then be determined by integrating this value using Equation (19), with the vitrification and saturation pressures serving as the upper and lower boundary conditions, respectively.

(19) $$N_{total}=\int_{P_{sat}}^{P_{vit}}N_{hom}\frac{dP}{dP/dt}$$

The vitrification of the PCL–CO_2_ mixture has been experimentally determined in numerous studies, providing accurate values. Consequently, the vitrification pressure at the gas absorption temperature of 313 K (4.81 MPa) was extracted from these studies and used as a boundary condition in the modeling. The data extracted from the literature were fitted to Equation (20), which was used to calculate $P_{vit\;}$ at 313 K [35,36].

(20) $$P_{vit}=-0.5578+(1.25\times 10^{15})\times e^{\left(-0.10569T\right)}$$

## 3. Results

In this study, we combined modified versions of the traditional CNT with numerical analysis to develop a modeling approach that closely aligns with experimental data. The numerical analysis method was coded in Python (Python Software Foundation V3.12.6, Wilmington, NC, USA); the code is provided in Supplementary S1. Users can input the material properties of a polymer and penetrant gas to calculate key foaming parameters (such as solubility, interfacial tension, energy barrier, and cell density). This approach can be applied to various polymers in addition to PCL. If the binary interaction parameter between the polymer and CO_2_ is experimentally determined, the provided Python template allows for easy calculation of solubility and cell density. This study presents the software implementation of a direct calculation method, previously unavailable in existing studies. This enables users to readily analyze cell morphology and apply the findings to their research requirements.

**Figure 3:**
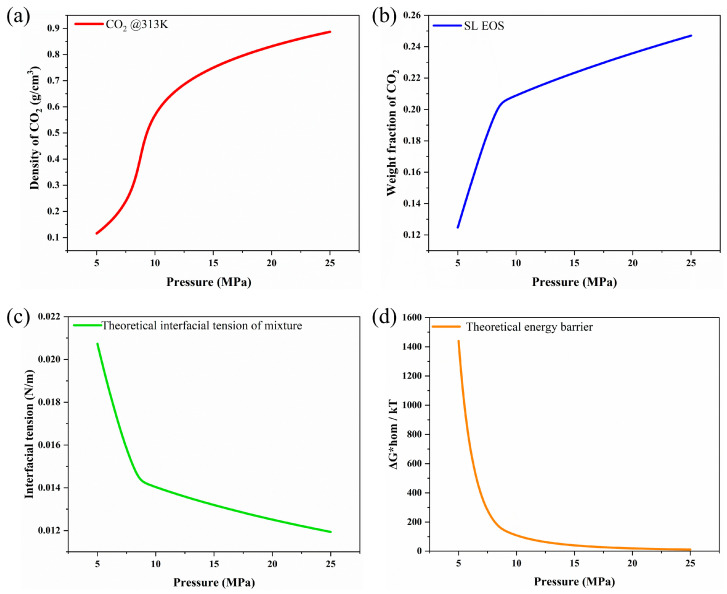
(**a**) CO_2_ density as a function of pressure at 313 K. Physical parameters to calculate cell density at 313 K: (**b**) CO_2_ solubility, (**c**) interfacial surface tension, and (**d**) cell nucleation energy barrier.

**Figure 4:**
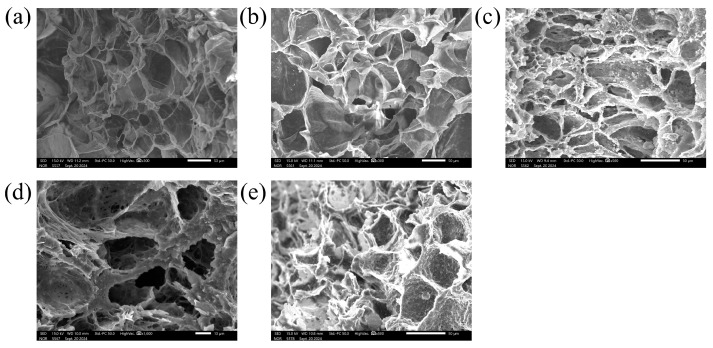
SEM images of PCL samples at 313 K under varying saturation pressures and depressurization rates. (**a**) 6 MPa, −0.3 MPa/s, ×300, (**b**) 7 MPa, −0.3 MPa/s, ×300, (**c**) 8 MPa, −0.3 MPa/s, ×500, (**d**) 9 MPa, −0.3 MPa/s, ×1000, and (**e**) 8 MPa, −1 MPa/s, ×500.

**Figure 5:**
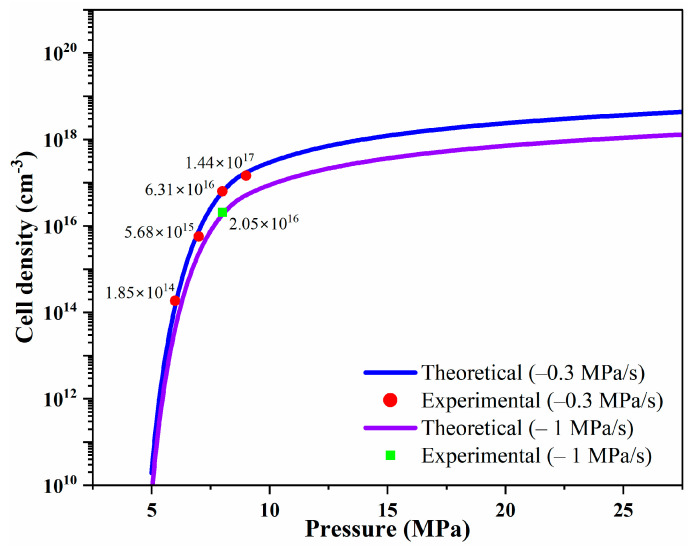
Theoretical cell density at 313 K predicted by SL-EOS and CNT, compared with experimentally measured cell density at 6, 7, 8, and 9 MPa, 313 K, −0.3, MPa/s and 8 MPa, 313 K, −1 MPa/s.

The MCP was conducted using depressurization during solid-state batch foaming. As shown in Figure 2, CO_2_ absorption occurred at 6~9 MPa and 313 K, followed by depressurization at a rate of −0.3 and −1 MPa/s to induce cell nucleation and foaming. The accuracy of the model was validated by comparing its results with experimental data, leading to the identification of coefficients that matched the experimental observations. The CO_2_ solubility, calculated using the SL-EOS and PRVS-EOS, was compared with reference data and was found to be consistent with the experimental values (Figure 3b). Figure 3a depicts the density of CO_2_ at 313 K as a function of pressure, calculated using the PRVS EOS. Figure 3c presents the interfacial surface tension of the PCL–CO_2_ mixture, which decreases with increasing batch chamber pressure. Figure 3d illustrates the Gibbs activation energy barrier, which also decreases with rising pressure. Finally, cell density was calculated based on the previously determined values and measured using the SEM images (Figure 4) with the results presented in graph form in Figure 5. This graph displays both the cell densities obtained from modeling and experimental data. The experimental cell density was determined by applying the MCP to PCL, followed by fracturing the sample with liquid nitrogen (N_2_; 77 K). The fractured cross sections were examined using field-emission scanning electron microscopy (FE-SEM; JSM-7001F, JEOL Ltd., Tokyo, Japan) and the SEM image can be found in Figure 4. By observing the SEM images in Figure 4a–d, it can be seen that although the magnitudes of each image are different, the cell density increases. Additionally, Figure 4e shows that at a saturation pressure of 8 MPa, the cell density is lower compared to Figure 4c, where the depressurization rate is 0.3 MPa/s. This indicates that in PCL, as the depressurization rate increases, there is a tendency for the cell density to decrease. The cell density was quantified from SEM images using ImageJ software (Version 1.8.0), and these experimental values were compared with the modeled results shown in Figure 5.

(21) $$Cell\;density\;({cm}^{-3})={\left(\frac{nM^{2}}{A}\right)}^{\frac{3}{2}}$$

Here, *n* denotes the number of bubbles in the micrograph, *M* represents the magnification factor of the micrograph, and *A* signifies the surface area of the micrograph.

Figure 5 compares the experimental values with the modeled values obtained using the Python simulation tool developed in this study. The results show a strong correlation between the experimental data and the theoretical modeling values. It was also observed that, for the same material, at a saturation pressure of 8 MPa, the values at depressurization rates of −0.3 MPa/s and −1 MPa/s share the same *A* and *B* factors.

## 4. Conclusions

This study proposes a novel method for predicting cell density pertaining to the MCP. The original model, which relied on CNT, often exhibited discrepancies with experimental values. We addressed this issue by incorporating the *A* and *B* factors. By adjusting the *A* and *B* factors for specific materials, predictions for other experiments can be made by conducting a few experiments. Furthermore, by validating the developed CNT-based modeling with the experimental results, we were able to demonstrate that the model proposed in this study aligns well with the experimental data, confirming its accuracy and reliability.

Although there are limitations due to the varying A and B factors when using PCL with different compositions from various manufacturers, these challenges can be addressed. If nucleation theory could be further established based on molecular structure or PCL composition, the simulation tool developed in this study could potentially be applied not only to all types of PCL but also to a wide range of polymers. This would enable accurate predictions of cell morphology, extending its applicability to various industries. Such advancements would allow for the precise production of microcellular foam products, whose behavior could be predicted more reliably across a wide range of industrial sectors.

Moreover, in the realm of biomaterials, this simulation tool could enable the creation of materials that maintain their structural integrity for a specific, pre-designed duration within the human body before dissolving. This would be particularly valuable for batch foaming processes, paving the way for innovative bioresorbable materials tailored to medical needs.

In this study, we used CO_2_, an inert gas, as the penetrant gas to calculate the cell morphology. However, in actual industrial settings, nitrogen (N_2_) is more commonly employed, as it allows for quicker polymer–gas mixtures. Therefore, if future research extends this work to predict and calculate the behavior of N_2_ and polymers, it will lead to an even faster industrial application, providing a practical and highly efficient solution for real-world manufacturing processes.

## Figures and Tables

**Table 1:** Experimental parameters of the MCP.

Property	Value
Saturation pressure (MPa)	6, 7, 8, 9 ± 0.2
Saturation temperature (K)	313 ± 2
Saturation time (min)	15
Depressurization rate (MPa/s)	−0.3, −1

**Table 2:** Specific properties for PRSV-EOS.

${P_{c}}^{*}\;(MPa)$	${T_{c}}^{*}$ (K)	ω	$\kappa_{1}$
7.382	304.2	0.225	0.04285

**Table 3:** Characteristic parameters for SL-EOS [20].

Subscript	Component	$P^{*}$	$T^{*}$	$\rho^{*}$	$v^{*}$	$r^{0}$
1	CO_2_	574.5	305.3	1.510	4.42	6.6
2	PCL	548.6	637.7	1.158	9.66	9.5
None	PCL–CO_2_	Can be calculated using Equations (1)–(13)				-

## Data Availability

The raw data supporting the conclusions of this article will be made available by the authors on request.

## Supplementary Materials

The following supporting information can be downloaded at https://www.mdpi.com/article/10.3390/polym16192723/s1: Python code S1: Cell morphology calculation code.
