# Supplementary material for: Modeling and Experimental Validation of Cell Morphology in Microcellular-Foamed Polycaprolactone
Source: Polymers (Basel). 2024 Sep 26;16(19):2723. doi: 10.3390/polym16192723 (PMC11478481; doi:10.3390/polym16192723)
Supplement: Supplementary file 1 [file polymers-16-02723-s001.zip › polymers-3183378-supplementary.pdf]

Supplementary

S1. Cell density calculation python code

```
import numpy as np
from scipy.integrate import quad
from scipy.optimize import fsolve
import matplotlib.pyplot as plt
import pandas as pd
import os

# Input data
T = 313  # K
T_CO2 = T
T_PCL = T
R = 8.314  # J/(mol·K)
k = 1.38 * 10**(-23)  # J/K
N = 6.02 * 10**23  # molecules/mole
P_vitr = 4.81 #MPa
A=10**-3.5
B=0.03 #B-factor
delta_P = -5 #depressurization rate (MPa/s)

# Sanchez-Lacombe parameters for PCL and CO2

#PCL properties
P_star_PCL = 548.6  # MPa
T_star_PCL = 637.7  # K
rho_star_PCL = 1.158  # g/cm^3
v_star_PCL = 9.66
r_zero_PCL = 9.5
```

```
rho_PCL = 1.16

#CO2 properties
M_CO2 = 44.01  # g/mol
P_star_CO2 = 574.5  # MPa
T_star_CO2 = 305.3  # K
rho_star_CO2 = 1.510  # g/cm^3
v_star_CO2 = 4.42
r_zero_CO2 = 6.6
r_CO2 = P_star_CO2 * M_CO2 / (R * T_star_CO2 * rho_star_CO2)

# Binary interaction parameters
psi = 0.98
X1 = 22.7 / (R * T)

# Parameters for CO2 PRSV EOS
T_c = 304.2  # K
P_c = 7.3824  # MPa
omega = 0.225
kappa_1 = 0.04285

#CO2 density calculation (PRSV EOS)
def CO2_density_PRSV(P, T):
    Tr = T / T_c
    kappa_o = 0.378893 + 1.4897153 * omega - 0.17131848 * omega**2 + 0.01965544 *
omega**3

    if Tr > 0.7:
        kappa = kappa_o
    else:
        kappa = kappa_o + kappa_1 * (1 + Tr**0.5) * (0.7 - Tr)
```

```
    alpha = (1 + kappa * (1 - Tr**0.5))**2
    a = alpha * 0.457235 * (R * T_c)**2 / P_c
    b = 0.077796 * R * T_c / P_c

    def equation(Vm):
        return (R * T) / (Vm - b) - a / (Vm * (Vm + b) + b * (Vm - b)) - P

    Vm_initial_guess = 30
    Vm_solution = fsolve(equation, Vm_initial_guess)

    if Vm_solution[0] > b:
        Vm = Vm_solution[0] / 1000
        rho = M_CO2 / Vm
        rho_CO2 = rho / 1000

    return rho_CO2

# Calculate rho_reduced
def calculate_rho_reduced(phi_CO2, T_reduced, P_reduced, r_CO2):
    # Initial guess for rho_reduced
    rho_reduced_initial = 0.8

    def equation6(rho_reduced):
        return 1 - np.exp(-((rho_reduced) ** 2) / T_reduced - P_reduced / T_reduced - (1 -
phi_CO2 / r_CO2) * rho_reduced) - rho_reduced

    # Iteratively solve for rho_reduced
    rho_reduced = fsolve(equation6, rho_reduced_initial)[0]
    return rho_reduced
```

```
# Calculate volume fraction of CO2
def equation7(phi_CO2_new, phi_CO2, rho_reduced, T_reduced, P_reduced, X1, r_CO2,
T_reduced_CO2, P_reduced_CO2, rho_reduced_CO2):
    if phi_CO2_new <= 0:
        return np.inf
    term1 = np.log(phi_CO2_new) + (1 - phi_CO2_new) + rho_reduced * M_CO2 /
rho_star_CO2 * X1 * (1 - phi_CO2_new) ** 2
    term2 = r_CO2 * (-rho_reduced / T_reduced_CO2 + P_reduced / (T_reduced_CO2 *
rho_reduced) +
                      (1 - rho_reduced) * np.log(1 - rho_reduced) / rho_reduced +
np.log(rho_reduced) / r_CO2)
    term3 = r_CO2 * (-rho_reduced_CO2 / T_reduced_CO2 + P_reduced_CO2 /
(T_reduced_CO2 * rho_reduced_CO2) +
                  (1 - rho_reduced_CO2) * np.log(1 - rho_reduced_CO2) / rho_reduced_CO2
+ np.log(rho_reduced_CO2) / r_CO2)
    return term1 + term2 - term3


def calculate_phi_CO2_new(phi_CO2, rho_reduced, T_reduced, P_reduced, X1,
r_zero_CO2, T_reduced_CO2, P_reduced_CO2, rho_reduced_CO2):
        return fsolve(equation7, phi_CO2, args=(phi_CO2, rho_reduced, T_reduced,
P_reduced, X1, r_zero_CO2, T_reduced_CO2, P_reduced_CO2, rho_reduced_CO2))[0]


# Iterative calculation to find the solution of volume fraction of CO2
def find_final_phi_CO2(P, initial_phi_CO2=0.0001, tolerance=0.0001, max_iter=10000):
    phi_CO2 = initial_phi_CO2
    error = 1
    iter_count = 0

    while error > tolerance and iter_count < max_iter:
        iter_count += 1
        phi_PCL = 1 - phi_CO2

        # Step 1: Calculate P_star_mix
          P_star_mix = phi_PCL * P_star_PCL + phi_CO2 * P_star_CO2 - phi_PCL *
```

```
phi_CO2 * (P_star_PCL + P_star_CO2 - 2 * psi * np.sqrt(P_star_PCL * P_star_CO2))

        # Step 2: Calculate T_star_mix
        T_star_mix = P_star_mix / (phi_PCL * P_star_PCL / T_star_PCL + phi_CO2 * P_star_CO2 / T_star_CO2)

        # Calculate reduced variables
        T_reduced = T / T_star_mix
        P_reduced = P / P_star_mix
        T_reduced_CO2 = T / T_star_CO2
        P_reduced_CO2 = P / P_star_CO2
        rho_reduced_CO2 = CO2_density_PRSV(P, T) / rho_star_CO2

        # Step 3: Calculate rho_reduced
        rho_reduced = calculate_rho_reduced(phi_CO2, T_reduced, P_reduced, r_CO2)

        # Step 4: Calculate phi_CO2_new
        phi_CO2_new = calculate_phi_CO2_new(phi_CO2, rho_reduced, T_reduced, P_reduced, X1, r_zero_CO2, T_reduced_CO2, P_reduced_CO2, rho_reduced_CO2)

        error = abs(phi_CO2 - phi_CO2_new)
        phi_CO2 = phi_CO2_new

    # Calculate w_CO2 (weight fraction of CO2) and check if w_CO2 + w_PCL = 1
    w_CO2 = phi_CO2 / (phi_CO2 + (1 - phi_CO2) * rho_star_PCL / rho_star_CO2)

    rho_CO2 = CO2_density_PRSV(P, T)

    # Calculate rho_star_mix
    w_PCL = 1 - w_CO2
    rho_star_mix = 1 / (w_CO2 / rho_star_CO2 + w_PCL / rho_star_PCL)
```

```
    # Calculate rho_mix (density of PCL-CO2 mixture)
    rho_mix = rho_reduced * rho_star_mix

    return phi_CO2, w_CO2, rho_CO2, rho_star_mix, rho_mix

# Calculate phi_CO2, w_CO2, rho_CO2, rho_star_mix, rho_mix, gamma_mix, delta_Gibbs_hom, C, and nucleation rate for a range of pressures
pressures = np.linspace(5, 30, 500) #Pressure range
phi_CO2_values = []
w_CO2_values = []
rho_CO2_values = []
rho_star_mix_values = []
rho_mix_values = []
gamma_mix_values = []
delta_Gibbs_hom_values = []
C_values = []
nucleation_rate_values = []

for P in pressures:
    phi_CO2, w_CO2, rho_CO2, rho_star_mix, rho_mix = find_final_phi_CO2(P)
    phi_CO2_values.append(phi_CO2)
    w_CO2_values.append(w_CO2)
    rho_CO2_values.append(rho_CO2)
    rho_star_mix_values.append(rho_star_mix)
    rho_mix_values.append(rho_mix)
    gamma_mix = 0.0456*(rho_mix/rho_PCL)**4*((1 - w_CO2)**4)  # N/m
    gamma_mix_values.append(gamma_mix)
    delta_Gibbs_hom = (16 * np.pi * gamma_mix**3) / (3 * ((P - 0.1) * (10**6))**2)  #
Nm=J
    delta_Gibbs_hom_values.append(delta_Gibbs_hom)
```

```
    # Calculate Concenctration
    C = w_CO2 * rho_PCL * N / (M_CO2 * (1 - w_CO2))  # number of molecules / cm3
    C_values.append(C)

    # Calculate Nucleation Rate
    nucleation_rate = (10**(-5))*A * C * np.exp(-delta_Gibbs_hom*B/(k * T))
    nucleation_rate_values.append(nucleation_rate)

cell_density_values = []

for i, P in enumerate(pressures):
    integrand = lambda x: nucleation_rate_values[i] / delta_P  # 적분할 함수 정의
    cell_density, error = quad(integrand, P, P_vitr)  # 적분 수행
    cell_density_values.append(cell_density)


# Plotting C and Nucleation Rate
fig2, ax2 = plt.subplots(1, 2, figsize=(12, 6))

ax2[0].plot(pressures, C_values, 'bo-')
ax2[0].set_xlabel('Pressure (MPa)')
ax2[0].set_ylabel('Concentration C')
ax2[0].set_title('Variation of Concentration C with Pressure')
ax2[0].grid(True)

# Plotting log(Nucleation Rate)
nucleation_rate_values = nucleation_rate_values
ax2[1].plot(pressures, nucleation_rate_values, 'ro-')
ax2[1].set_yscale('log')
```

```
ax2[1].set_xlabel('Pressure (MPa)')
ax2[1].set_ylabel('log(Nucleation Rate)')
ax2[1].set_title('Variation of log(Nucleation Rate) with Pressure')
ax2[1].grid(True)

fig2.tight_layout()
plt.show()

# Plotting Cell Density
plt.figure(figsize=(8, 6))
plt.plot(pressures, cell_density_values, 'mo-')
plt.yscale('log')
plt.xlabel('Pressure (MPa)')
plt.ylabel('Cell Density')
plt.title('Cell Density over Pressure')
plt.grid(True)
plt.show()

# Create a excel DataFrame with the desired values
data = {
    'Pressure (MPa)': pressures,
    'gamma_mix (N/m)': gamma_mix_values,
    'rho_CO2 (g/cm^3)': rho_CO2_values,
    'rho_mix (g/cm^3)': rho_mix_values,
    'w_CO2': w_CO2_values,
    'cell_density': cell_density_values,
    'delta_gibbs_hom/kT': delta_Gibbs_hom_values
}

df = pd.DataFrame(data)
```

```
# Save the DataFrame to an Excel file
df.to_excel('output_values1.xlsx', index=False)

print("Data has been successfully saved to 'output_values.xlsx'")
print(os.getcwd())
```
